# T-helper 1 versus T-helper 2 lymphocyte immunodysregulation is the central factor in genesis of Burkitt lymphoma: hypothesis

**DOI:** 10.1186/1750-9378-2-10

**Published:** 2007-05-17

**Authors:** Joseph Lubega

**Affiliations:** 1 Department of Paediatrics, County Durham and Darlington NHS Foundation Trust, Darlington, UK

## Abstract

**Background:**

The HIV epidemic has challenged our previous understanding of endemic Burkitt's lymphoma. Despite the strong association of Burkitt's lymphoma and HIV infection in the Developed world, and against previous postulations that the cancer is due to immunosupression among African children, the HIV epidemic in the Malaria belt has not been associated with a corresponding increase in incidence of childhood Burkitt's lymphoma. Even outside the context of HIV infection, there is substantial evidence for a strong but skewed immune response towards a TH2 response in genesis of Burkitt lymphoma.

**Presentation of the hypothesis:**

Rather than a global and/or profound immunosupression, the final common pathway in genesis of Burkitt's lymphoma is the dysregulation of the immune response towards a TH2 response dominated by B-lymphocytes, and the concomitant suppression of the TH1 cell-mediated immune surveillance, driven by various viral/parasitic/bacterial infections.

**Testing the hypothesis:**

Case control studies comparing TH2 and TH1 immune responses in Burkitt lymphoma of different etiological types (sporadic, HIV-related, endemic and post-transplant) to demonstrate significant dominance of TH2 immune response in presence of poor CMI response as a common factor. Immunological profiling to evaluate differences between immune states that are associated (such as recurrent Malaria infection) and those that are not associated (such as severe protein-energy malnutrition) with Burkitt lymphoma. Prospective cohorts profiling chronology of immunological events leading to Burkitt lymphoma in children with EBV infection.

**Implications of the hypothesis:**

The dysregulation of the immune response may be the missing link in our understanding of Burkitt lymphomagenesis. This will provide possibilities for determination of risk and for control of development of malignancy in individuals/populations exposed to the relevant infections.

## Background

Burkitt lymphoma represents the first strong association of cancer and viral infection – Epstein – Barr virus [1]. The striking endemicity, different epidemiological forms, fast progression and peculiar dramatic clinical presentation of Burkitt lymphoma still make it an invaluable tumor for studying oncogenesis [2,3]. Yet less than enough effort has been put in elucidating the immunological and cytogenetic mechanisms of genesis of this tumor.

The exact contribution of EBV to BL genesis remains controversial. There is evidence that EBV infection causes excessive B-cell proliferation by chronic stimulation of immunoglobulin genes [4]. The mode of stimulation has been suggested to be by causing expression of somatic hypermutation inducing molecules – including activation-induced cytidine and polymerase-eta [5]. Ultimately accrual of oncogenic mutations occurs. Whether and how EBV contributes to cytogenetic lesions including *c-myc *reciprocal translocations, *bcl-6 *mutations and the universal *p53 *mutation is not certain [6]. It is suggested that these cytogenetic lesions may be constitutive, as a result of B-cell hyperproliferation. Though EBV does immortalize B-cells in vitro to form lymphoblastoid cell lines, these LCLs are phenotypically distinct from Burkitt's tumor cells [7,8] – suggesting other in vivo factors play part in Burkitt's malignant transformation. The phenotype of the EBV-transformed B-lymphocytes suggests that the effect of viral protein expression mimics that of antigen-driven lymphocyte activation [9]. It is also worth noting that EBV infection is not a necessity to genesis of BL, though present in a vast majority of cases.

Malaria and malnutrition, both very common in Equatorial Africa, are thought to play part in the immunological events leading to lymphomagenesis [10-12]. Specifically, Malaria is known to alter cytokine production resulting in polyclonal B cell activation and impairment of EBV-specific T cell responses [13,14]. Recent evidence also suggests that Malaria facilitates EBV replication [15]. However, neither Plasmodium *falciparum *nor Epstein-Barr virus infection has been associated with increased detection of chromosomal translocations [15,16].

With that background understanding of the genesis of Burkitt lymphoma and the nature of interaction of tumor, Epstein – Barr Virus and Malaria, it would be logical that a "Burkitt tumor epidemic" follows the HIV/AIDS epidemic in Africa. HIV infection would be expected to act as catalyst to Burkitt lymphomagenesis. In fact the incidence of Burkitt Lymphoma in Europe/North America has exponentially risen since the advent of HIV [18-20]. However, even in the Western setting, BL has been demonstrated to occur in the early stages of HIV infection when immunosupression is mild or even absent [21-24].

Even before the HIV/AIDS era there were observations suggesting selective suppression and enhancement of different aspects of the immune response in patients with Burkitt Lymphoma. Though there are no studies directly correlating this, anecdote indicates that the tumor is not a feature of severely malnourished (e.g. those with Kwashiorkor/Marasmus) African children. In the context of HIV-infection, B-cell stimulation (indicated by higher serum globulin) is an *independent predictor *of NHLs and precedes the diagnosis of NHL by several years [22]. Severe post-chemotherapy immunosupression is also not associated with neo- or relapse of Burkitt's tumor [25]. Though of B-cell origin, post-transplant lymphoproliferative disorders are also predominantly of non-Burkitt phenotype usually diffuse large B-cell lymphomas [26,27].

### HIV infection and Burkitt lymphoma in Africa

Human Immunodeficiency virus is the most effective biological immunosupressant known to man. As such, it has resulted in a cancer epidemic – in Africa – and worldwide. HIV infection has been confirmed to favor production of cytokines IL-4 and IL-10 resulting in a TH2 dominant immune profile, which enhances B-cell proliferation [28,29].

HIV/AIDS-associated haematological malignancies tend to be particularly of B-cell origin; have a tendency to present in extra nodal sites, rapid clinical progression, and are associated with Epstein-Barr virus (EBV) infection [18]. Burkitt lymphoma therefore has all features typical of HIV-associated lymphomas. Paradoxically, since the advent of the HIV epidemic almost all studies in Sub-Saharan Africa have consistently demonstrated no increased incidence of BL in general, nor among children with HIV infection specifically.

Chifumbe Chintu et al, 1995 in Zambia, compared histopathological records 1980 – 1982 vs. 1990 – 1992, at University Teaching Hospital, Lusaka [30]. They demonstrated that the [reported] incident cases of Burkitt lymphoma significantly reduced: 18 vs. 11 (p = 0.05) in the HIV/AIDS era. Other clinical and demographic characteristics of Burkitt lymphoma remained unchanged.

A case series in Zimbabwe in 1998 studied 76 consecutive cases of all newly diagnosed childhood cancer over a 6-month period at The Paediatric Oncology Unit, Parirenyatwa Teaching Hospital [31]. Despite a very high HIV seroprevalence rate in the study group – 27 out of 64 children assessable for HIV serology were HIV positive, a seroprevalence of 42.2% – no cases of Burkitt lymphoma were seen in all the 76 cases; most of them were Kaposi's sarcoma and other Non-Hodgkin's lymphomas, mainly Large cell.

The only study that suggested increased incidence of BL in HIV-infected children was a case-control study investigating HIV infection & Cancer in Kampala Uganda 1998 [32]. The study showed a 5-fold increase of Burkitt lymphoma in HIV – infected children. 30% (10/33) HIV cases vs. 6% (11/190) controls. (OR = 7.5, 95% CI 2.8 – 20.1, based on 33 cases of Burkitt lymphoma; p = 0.0001.) However, this study noted the high likelihood of misdiagnosis [on light microscopy] of other types of Non-Hodgkin's lymphoma common in HIV infection as Burkitt lymphoma. Other studies including by *Mbidde et al*. in Uganda, *Lazzi et al*. in Kenya, *Sinfield et al*. in Malawi and *Lucas et al*. in Côte d'Ivoire [33-36] did not find a positive correlation between Burkitt lymphoma and childhood HIV infection.

Perhaps the most robust study that has investigated this issue was a case-control study done in Kampala Uganda in 1999, investigating Non-Hodgkin's lymphomas and HIV infection with age/sex matched controls, and their relationship with EBV infection [37]. 132 children with a mean age of 7 years were histologically diagnosed with Burkitt lymphoma; of which 61 had tissues validated and phenotyped confirming 56 as BL. HIV prevalence was 5.4% (3/54) in cases, compared with 5.0% (11/194) in controls, corresponding to an OR of 1.0 (0.3–3.9). All (51/51) cases of BL were EBV positive.

Therefore, evidence so far has demonstrated no positive association of the HIV epidemic and incidence of Burkitt lymphoma in sub-Saharan Africa. Why are African children with HIV infection NOT prone to Burkitt tumour? A simplistic explanation has been that the survival of African children with HIV infection is so poor that they do not leave long enough to have [clinical] Burkitt lymphoma [37]. This however is questionable. If immunosupression were the key major event leading to BL genesis, and knowing that EBV infection occurs early in childhood, and that EBV and HIV enhance each other, why doesn't HIV infection hasten development of BL? Why do the children instead suffer primary CNS and Non-Burkitt Non-Hodgkin lymphomas? I propose an alternative hypothesis taking all the above observations into account.

## Presentation of the hypothesis

Considering the above epidemiological observations and the current evidence regarding the cytogenetic events following chronic EBV infection, immune-dysregulation seems to be the key/ultimate factor in causation of BL: A prolonged hyperproliferation of B-cells (which predisposes them to cytogenetic lesions) stimulated by EBV; in an individual with dominance of TH2 immune response cytokines; and the subsequent and/or concurrent suppression of the TH1 immune response resulting in inefficient tumour cellular surveillance, is the cause of Burkitt lymphoma. A global (Humoral and Cell-mediated) immunosupression or a depressed TH2 and TH1 immune profile, may be "protective" against development of Burkitt lymphoma.

**Figure 1:**
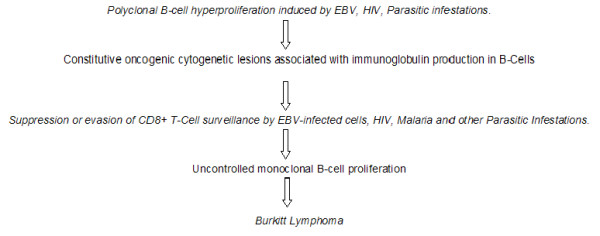
A schematic summary of the TH1/TH2 immunodysregulation hypothesis of Burkitt lymphomagenesis.

The hypothesis is consistent with the concept that oncogenic cellular lesions that result in cancers may be a consequence of an adaptive response to biologic stresses [38]. It highlights the polyclonal hyperproliferation of B-cells that precedes genetic mutations leading to monoclonal proliferation in BL; the permissive role of T-cell surveillance failure; the over-determination and the multifactorial infectious aetiology of BL [16]. It emphasizes that a TH2-dominant immune profile serves a duo purpose of propagating B-cell proliferation and suppressing T-cell function at the same time. The hypothesis also explains how EBV, HIV, Malaria and other Tropical infections act synergistically in genesis of BL. Figure 1 below summarizes the role of these infections.

The above hypothesis is consistent with the observation that Burkitt lymphoma is less common in individuals with profound and/or global suppression of both B-cell and T-cell mediated immune responses including childhood HIV/AIDS in the African setting, adults with advanced HIV/AIDS in the Western setting and in post-transplant lymphoproliferative disorders [22,26,37,39].

Children with severe protein-energy malnutrition seem to be protected from Burkitt lymphoma. Profound nutritional deficits (less than 5% protein per total daily food intake) depress both cellular and humoral immunity [40]. Other studies on the modulation of the immune response by moderate to severe chronic malnutrition have demonstrated a suppression of B-cell immunity, and enhancement of several aspects of T-cell immunity [41]. Several animal laboratory studies have also confirmed this pattern [42,43] – which (according to the hypothesis) is the reverse of the recipe required for Burkitt lymphomagenesis.

### The following study designs will test the hypothesis

▪ Case control studies examining blood samples of BL patients of various aetiological types (endemic, Sporadic, HIV-related and post-transplant) to demonstrate a dominant TH2 immune response in presence of poor CMI response as a common final pathway.

▪ Matched Case Control studies particularly profiling TH1/TH2 immune responses in immune states associated with BL (such as recurrent Malaria and early HIV infection) compared with immune states that are not associated with BL (such as severe Protein-Energy Malnutrition and late HIV infection).

▪ Prospective cohorts evaluating the role of TH1/TH2 immune profiles in modification of risk of BL among children with HIV/AIDS on HAART, in children with and who survive severe Protein-Energy malnutrition, and in areas of changing Malaria and other tropical infection endemicity.

## Implications of the hypothesis

Very few cases of oncogenic infections ever progress to malignancy. If dysregulation of the immune profile is the main event that leads to BL (or even other infectious cancers), determining the exposed individuals' immune profiles can be used to predict their likelihood of developing malignancy before they manifest clinical disease.

If the endpoint in BL genesis is TH2/TH1 immune-dysregulation, several infectious/environmental factors (other than EBV, Malaria, HIV) that contribute to lymphomagenesis, both in endemic and sporadic cases, may be identified and mitigated to modify the risk for malignancy.

The proposition that a B-cell malignancy such as BL may result from "disorganization" of the immune response challenges us to formulate immunotherapeutic strategies that will selectively target endogenous immunomodulators especially in preventing disease relapse.

## Abbreviations

**NHL **– Non-Hodkin's Lymphoma, **HIV **– Human Immunodeficiency Virus, **EBV **– Epstein-Barr Virus, **BL **– Burkitt Lymphoma, **TH2 **– T-Helper 2 lymphocytes, **TH1 **– T-Helper 1 lymphocytes.

## Competing interests

The author(s) declare that they have no competing interests.
